# Supplementary material for: Molecular basis of human CD22 function and therapeutic targeting
Source: Nat Commun. 2017 Oct 2;8:764. doi: 10.1038/s41467-017-00836-6 (PMC5624926; doi:10.1038/s41467-017-00836-6)
Supplement: Supplementary file 1 — Supplementary Information [file 41467_2017_836_MOESM1_ESM.pdf]

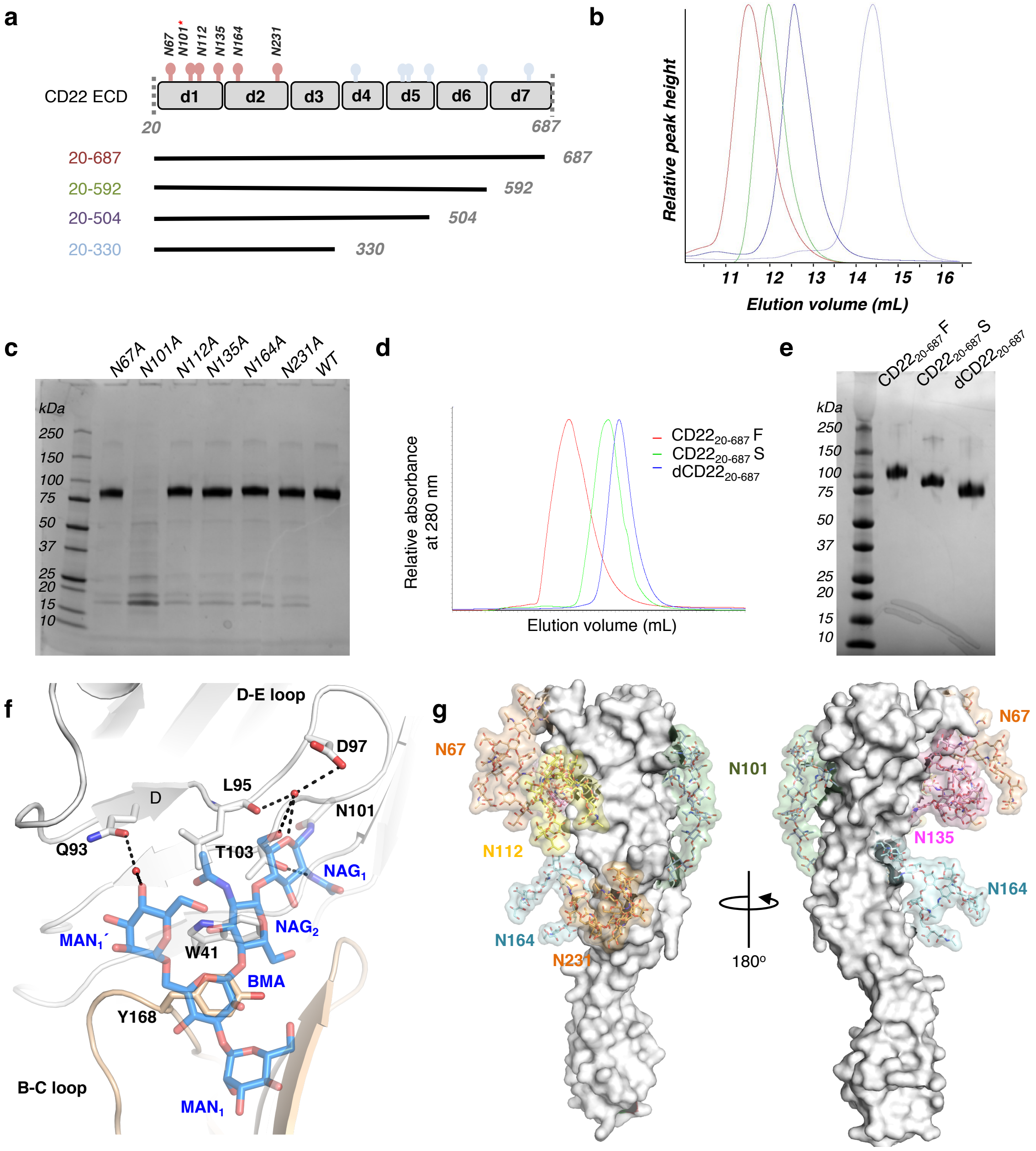

Supplementary Figure 1. Constructs of the CD22 extracellular domain (ECD) and associated N-linked glycans. (a) Schematic representation of the ECD of CD22 and constructs used for functional and structural studies. The secretion signal peptide (residues 1-19) is not represented. CD22 ECD is composed of seven Ig domains (d1-d7). Predicted N-glycosylation sites are depicted as sticks. (b) Superposition of size-exclusion chromatograms for 20-330 (grey), 20-504 (blue), 20-592 (green) and 20-687 (red) constructs. All constructs were monomeric in solution by size-exclusion chromatography. (c) N-linked glycosylation mutants for the $CD22_{20\text{-}687}$ (ECD) construct were evaluated by SDS-PAGE after expression in HEK293 Gnt $I^{-/-}$ mammalian cells. The N101A mutant showed no expression. (d) $CD22_{20\text{-}687}$ was expressed in two different cell lines: HEK293F cells are WT in their N-linked glycosylation machinery

($CD22_{20\text{-}687}$F), and HEK293 Gnt $I^{-/-}$ cells are mutant leading to N-linked glycosylation sites containing high-mannose glycans ($CD22_{20\text{-}687}$S). $CD22_{20\text{-}687}$S can be further treated with the enzyme EndoH, which results in the cleavage of high mannose glycans, retaining a single monosaccharide GlcNAc moiety at each N-linked glycan site (d$CD22_{20\text{-}687}$). (d) Overlaid size exclusion chromatograms of $CD22_{20\text{-}687}$F (red), $CD22_{20\text{-}687}$S (green), and d$CD22_{20\text{-}687}$ (blue) indicating a smaller size for the deglycosylated glycoprotein compared to its fully-glycosylated counterpart. (e) SDS-PAGE gel of $CD22_{20\text{-}687}$F, $CD22_{20\text{-}687}$S and d$CD22_{20\text{-}687}$ showing sample purity and size. (f) Interactions of the CD22 N-linked glycan 101 with the d1/d2 interface as observed in the $CD22_{20\text{-}330,5A}$ crystal structure. NAG_1 nitrogen H-bonds (black dashed lines) with the T103 side chain located in d2 loop D-E. A water-mediated H-bond is formed between NAG_1, the D97 side chain and the L95 main-chain carbonyl located in loop D-E. Moreover, W41 located in the d1 strand B makes van der Waals contacts with NAG_2. BMA stacks against the aromatic side chain of Y168 located in d2 loop B-C. Man_1' H-bonds via a water molecule with the Q93 side chain. (g) $CD22_{20\text{-}330}$ structure modelled with energy-minimized $GlcNAc_2FucMan_3GlcNAc_2Gal_2Sia_2$ glycans at all putative N-linked glycosylation sites [1]. One face is heavily glycosylated with five putative N-linked glycosylation sites, while the N101 glycan (essential for folding) is the only one localized on the opposing face.

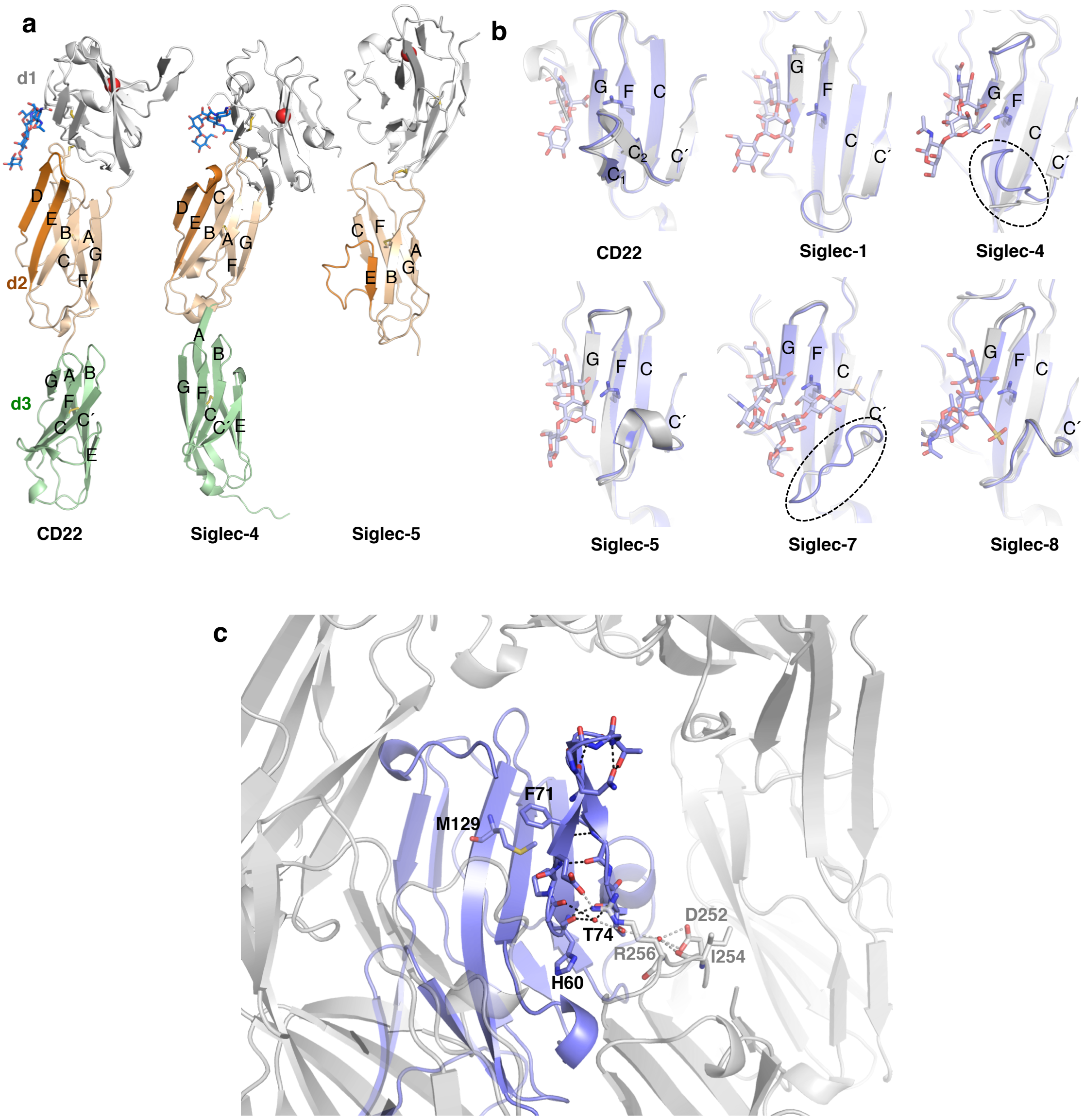

Supplementary Figure 2. Differences among Siglecs in d2 and sialic acid ligand binding site. (a) Crystal structures of CD22 and Siglec-4 (PDB ID: 5LFR) [2] show the relative position adopted by the N-terminal Ig domains d1 (grey), d2 (wheat) and d3 (green). d2 belongs to the C1-type Ig domain fold in CD22 and Siglec-4. The β-sheets of CD22 and Siglec-4 d2 are formed by strands ABED and CFG (C1-type fold), which is different from the predicted classic seven-stranded C2-type fold formed by ABE and C′CFG strands. Conversely, d2 in Siglec-5 (PDB ID: 2ZG2) [3] adopts a C2–type fold. d3 in CD22 and Siglec-4 adopts a C2-type fold. The conserved R120 for Sia binding is shown as a red sphere in d1. The conserved N-linked glycan in d1 that interacts with the d1/d2 interface is shown as sticks (blue). (b) Superposition of the unliganded (grey) and liganded (blue) structures of d1 from CD22, Siglec -1 (PDB ID: 1QFP and 1QFO) [4], -4 (PDB ID: 5LFR and 5LF5) [2], -5 (PDB ID: 2ZG2 and 2ZG3) [3], -7 (PDB ID: 1O7S and 2HRL) [5,6], and -8 (PDB ID: 2N7A and 2N7B) [7]. Overall, unliganded and complex structures of CD22, Siglec -1, -5 and -8 are highly similar. Loop C-C′ is disordered in unliganded Siglec -4 and -7 and becomes ordered upon ligand binding. Glycan ligands are represented as blue sticks in the binding pocket. (c) Interactions between the d1 domain (in blue) with symmetry related molecules (in grey) observed in the crystal of $CD22_{220\text{-}330,5A}$. The extensive intra-molecular H-bonds (black dots) between strands C1

and C2 primarily contribute to the pre-disposed nature of the CD22 ligand binding site. The conformation of the C_1/C_2 β-hairpin is also stabilized by van der Waals interactions between residue F71 located in strand C2 and M129 located in strand G. The base of the C_1/C_2 β-hairpin is involved in few H-bonds (grey dots) and water (red spheres) mediated H-bonds (grey dots) with residues in d3 from the symmetry related molecule.

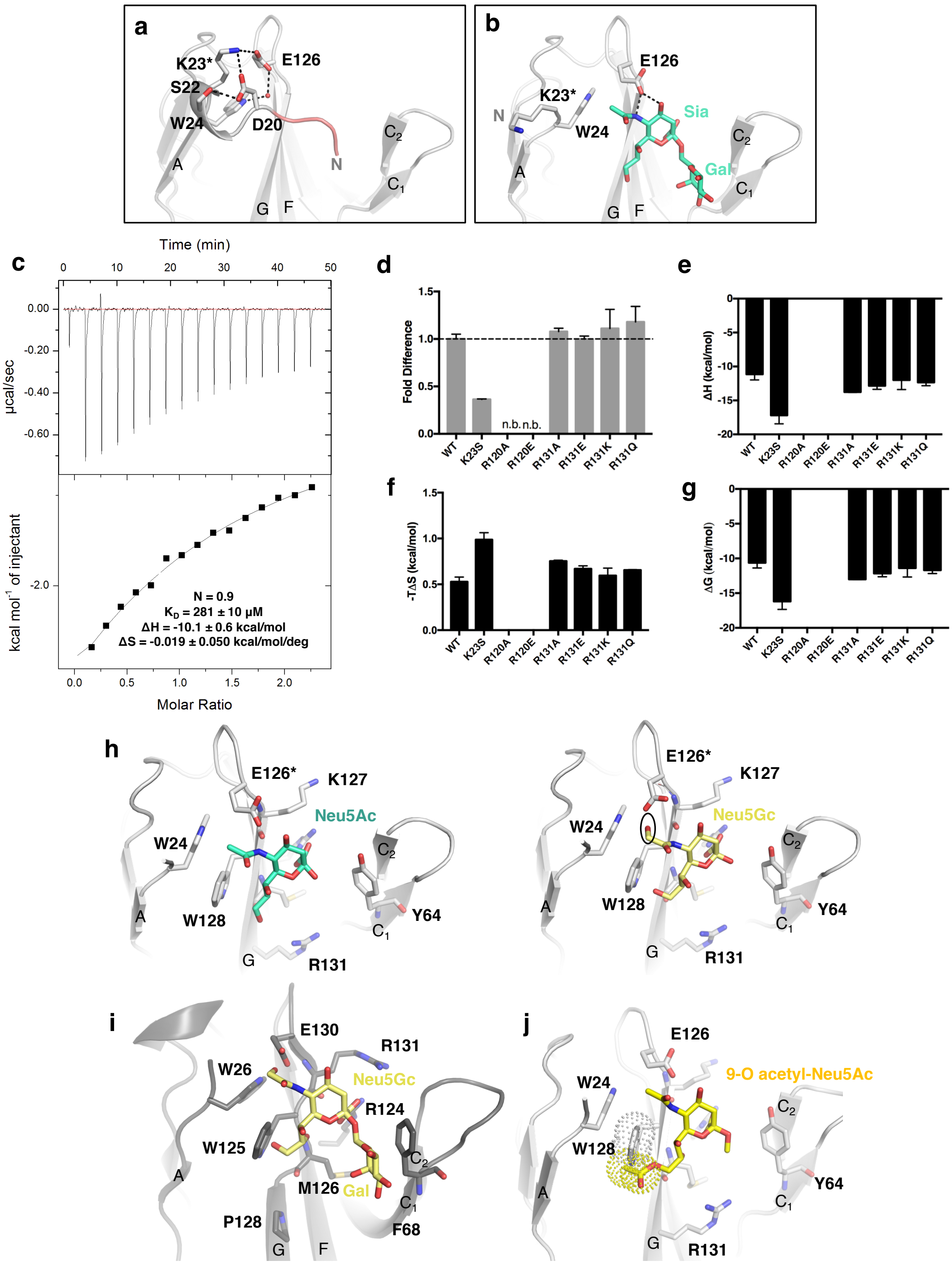

Supplementary Figure 3. Binding of α2-6 sialyllactose to CD22 and identification of important CD22 residues for binding. (a) In the unliganded state, residues 21-24 of CD22 adopt an α-helical turn, while residues 17-20 (in pink) are extended and occupy the binding pocket. The N-terminal α-helix is stabilized by salt bridges formed between the ε-amino group of K23 and the carboxyls of E126 (loop F-G) and D20 (N terminus). Mutation K23S located in the N-terminal portion improved the K_D of CD22 for α2-6 sialyllactose by 2.5-fold, likely by breaking the salt bridge with E126 in the unliganded

structure and by providing a free E126 carboxylate to interact with the N-acetyl moiety of Sia. (b) Binding of α2-6 sialyllactose displaces N-terminal residues 17-22 from the binding site, promoting the H-bond between E126 and Sia. Residues 17-19 in our crystallized constructs are a result of cloning overhangs and are in fact not native to CD22. It is unclear whether this observation is biological relevant or an artifact of our *in vitro* structure. (c) ITC data obtained by injecting α2-6 sialyllactose into a solution of WT $CD22_{20\text{-}330}$ at 25°C. N, stoichiometry; ΔH, change in enthalpy; −TΔS, change in entropy reported for duplicate measurements. (d) Binding affinities of CD22 mutants for α2-6 sialyllactose determined by ITC. K_D values for mutants to α2-6 sialyllactose are normalized with respect to that of WT $CD22_{20\text{-}330}$ (set to 1.0). Error bars represent the standard error of the mean (SEM) derived from two independent measurements and n.b. represents no detectable binding at the test concentrations. ΔH (e), −TΔS (f) and ΔG (g) are reported for the mutants. (h) Crystal structure of $CD22_{20\text{-}330,5A}$ bound to Neu5Ac (left) and modelled with Neu5Gc (right). In our modelling, the additional hydroxyl at position 5 in Neu5Gc (black circle) favors a more stable conformation for the E126 carboxylate, which in-turn H-bonds with W24. (i) Model of the mouse CD22 V-domain based on the crystal structure of human $CD22_{20\text{-}330,5A}$ and putative interactions with Neu5Gc(α2,6)Gal as generated by Molecular Operating Environment (MOE) [8]. In the mouse CD22 model, F68 in the C1/C2 ß-hairpin dictates the specificity for α2,6 glycans. P128 (R131 in human) does not H-bond with the C2 galactose hydroxyl in comparison to the human ortholog. (j) Model of the interaction between CD22 and 9-O-acetylated Neu5Ac reveals putative steric clashes (highlighted with dots) between the side chain of W128 and the acetyl group at position 9 in Sia.

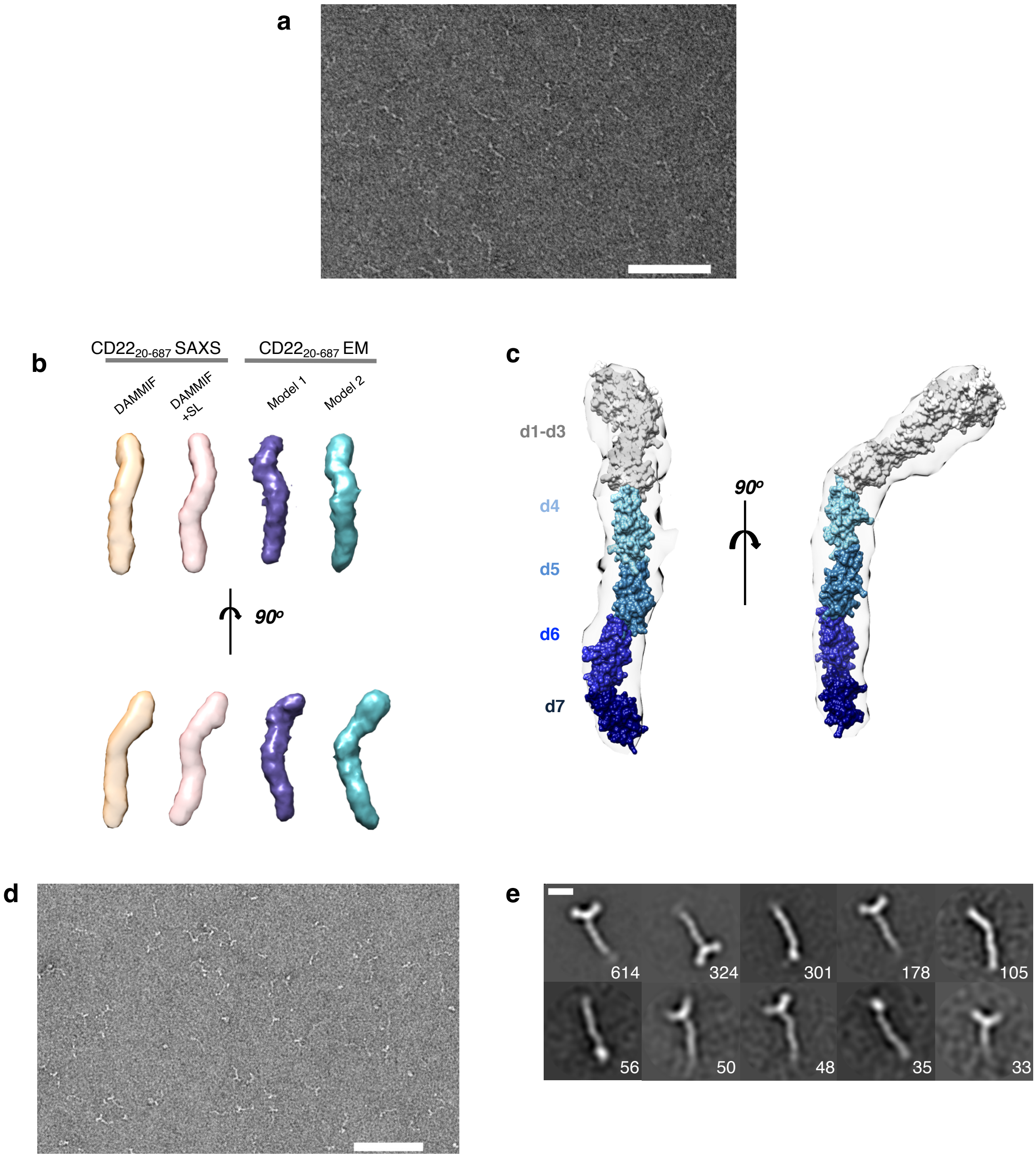

Supplementary Figure 4. EM and SAXS analysis of unliganded CD22$_{20\text{-}687}$ and CD22$_{20\text{-}687}$ + epratuzumab Fab. (a) Representative micrograph of negatively stained CD22$_{20\text{-}687}$ particles. Scale bar represents 100 nm. (b) CD22$_{20\text{-}687}$ SAXS envelopes generated with DAMMIF [9] (dark yellow); CD22$_{20\text{-}687}$ + α2-6 sialyllactose SAXS envelope generated with DAMMIF [9] (pink); two *ab initio* models generated with cryoSPARC [10] (purple and teal). (c) Fitting of Ig domain models into the negative stain EM envelope. The crystal structure of CD22$_{20\text{-}330,5A}$ (d1-d3) is shown in grey, while modelled C2-type domains d4-d7 are shown as shades of blue. (d) Representative micrograph of negatively stained CD22$_{20\text{-}687}$ + epratuzumab Fab particles. Scale bar represents 100 nm. (e) Best ten 2D class average images describing particles used as input for *ab initio* reconstruction of CD22$_{20\text{-}687}$ + epratuzumab Fab complex. Scale bar represents 10 nm. The number of particles per class is indicated.

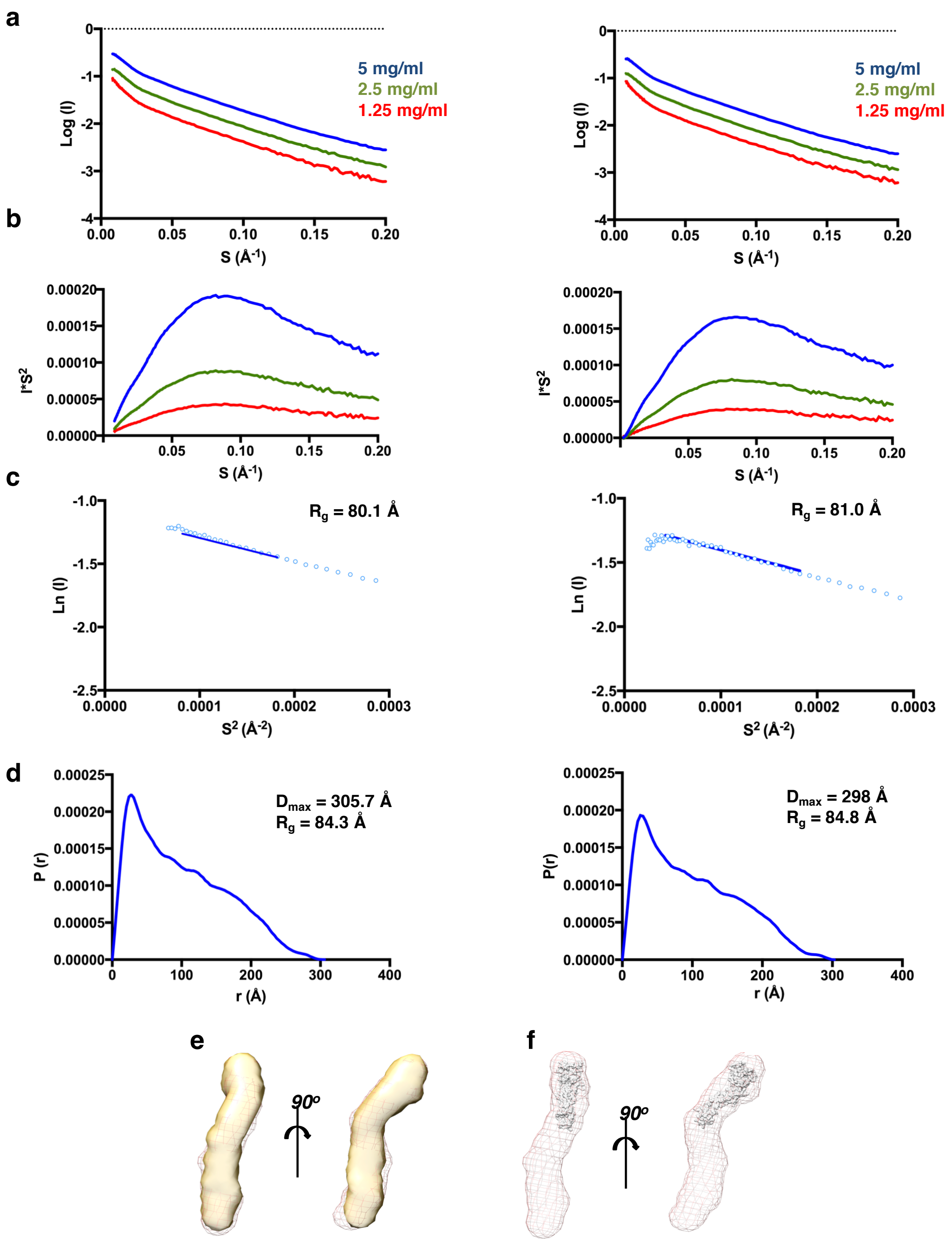

Supplementary Figure 5. Concentration dependence of SAXS measurements for CD22$_{20\text{-}687}$ in absence (left panels) and in presence (right panels) of α2-6 sialyllactose. (a) Solution scattering and (b) Kratky plots at 5 mg ml^{-1} (blue), 2.5 mg ml^{-1} (green) and 1.25 mg ml^{-1} (red). The highest concentration was selected for CD22$_{20\text{-}687}$ (left) and CD22$_{20\text{-}687}$ + α2-6 sialyllactose (right) for further analysis. (c) Guinier plot and (d) pair-distribution function (P(r)). (e) Superposition of the SAXS 3D volumes of CD22$_{20\text{-}687}$ (represented as a yellow surface) and CD22$_{20\text{-}687}$ complexed with α2-6 sialyllactose (red mesh). (f) The crystal structure of CD22$_{20\text{-}330,5A}$ is represented as surface (grey) and superimposed onto the SAXS volume obtained with the CD22$_{20\text{-}687}$ + α2-6 sialyllactose sample, illustrating that α2-6 sialyllactose binding to CD22$_{20\text{-}687}$ does not induce conformational changes in solution.

a

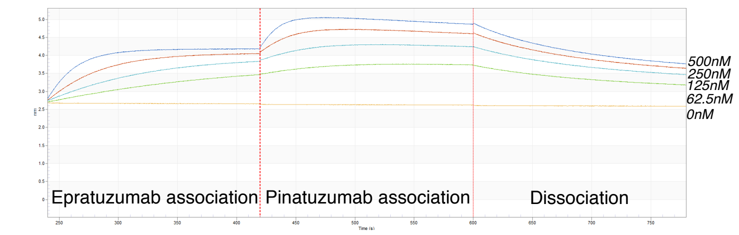

b

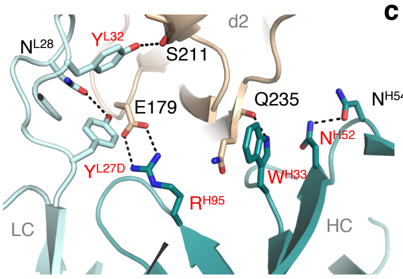

c

	K_D (µM)
WT	0.12 ± 0.008
$W^{H33}A$	no binding
$N^{H52}A$	0.26 ± 0.006
$R^{H95}A$	no binding
$Y^{L27D}A$	1.70 ± 0.14
$Y^{L32}A$	no binding

d

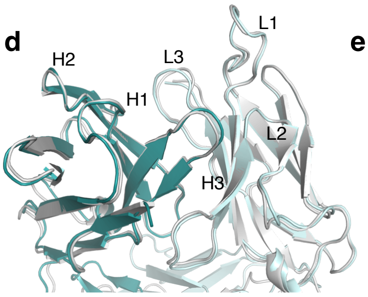

e

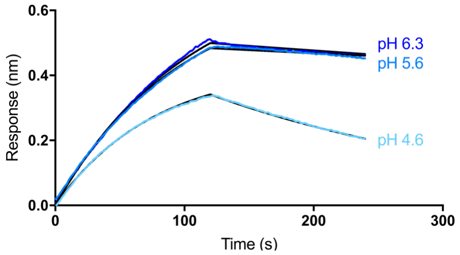

Supplementary Figure 6. Characterization of epratuzumab and pinatuzumab epitopes on $CD22_{20\text{-}687}$ by binding kinetics. (a) Biolayer interferometry competition experiments showing that both pinatuzumab and epratuzumab are capable of sequentially binding to CD22 without impacting their respective affinities. This indicates that pinatuzumab and epratuzumab bind to distinct, non-overlapping epitopes on CD22. (b) Mutated residues within the epratuzumab paratope based on the $CD22_{20\text{-}330,4Q}$ + epratuzumab Fab crystal structure (LC, light cyan; HC, deep teal) are shown as sticks with red labels. Interacting residues within the CD22 d2 structure are shown as wheat sticks. (c) Calculated K_D values of epratuzumab mutants binding to $CD22_{20\text{-}687}$ as determined by biolayer interferometry. Alanine point mutants of residues W^{H33}, R^{H95}, and Y^{L32} resulted in a complete loss of detectable affinity to CD22 under experimental conditions indicating an integral role for these residues in CD22 engagement. N^{H52} and Y^{L27D} retained the ability to engage CD22, but at a 2-fold and 12-fold decreased affinity, respectively. (d) The epratuzumab variable domains from the unliganded (grey) and the CD22-bound (dark and light cyan) crystal structures are superposed. No significant changes are observed in CDR conformations (r.m.s.d. of 0.50 Å), indicating that epratuzumab is in a predisposed conformation ideal for CD22 binding. (e) Binding kinetics of epratuzumab to CD22ectoF was measured by biolayer interferometry at pH 4.6, 5.6, and 6.3. Comparable affinity, k_{on} and k_{off} values were obtained for pH 5.6 and 6.3, whereas pH 4.6 showed a 10-fold faster off-rate, but still bound tightly.

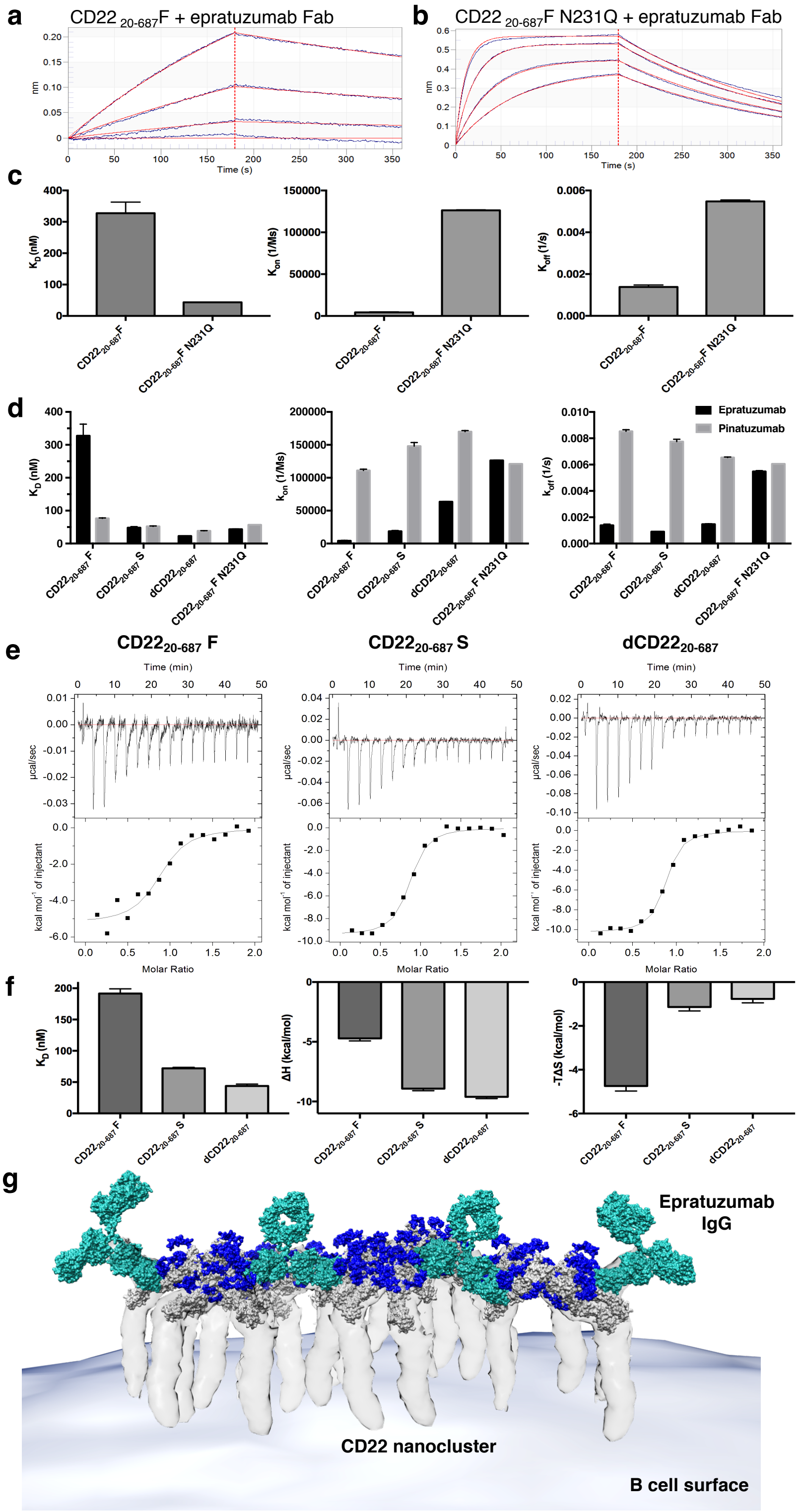

Supplementary Figure 7. Role of N-linked glycans in epratuzumab binding to CD22. (a) Fitted binding kinetics data of epratuzumab Fab association and dissociation to immobilized $CD22_{20\text{-}687}F$. (b) Fitted binding kinetics data of epratuzumab Fab association and dissociation to immobilized $CD22_{20\text{-}687}F$ N231Q. (c) Calculated K_D, k_{on} and k_{off} values for epratuzumab Fab binding to WT and N231Q $CD22_{20\text{-}687}F$. $CD22_{20\text{-}687}F$ N231Q displays a 6-fold higher K_D for epratuzumab Fab, primarily due to a ~25-fold improvement in k_{on}. This data indicates that the N231 glycan plays a steric role in

impeding epratuzumab access to its epitope on CD22, consistent with our crystal structure. Error bars represent SEM derived from three independent measurements. (d) Summary of epratuzumab and pinatuzumab binding to all CD22 tested glycoforms, including the N231Q glycan mutant. The size of N-linked glycans impacts binding by both antibodies; however, epratuzumab shows a much lower binding affinity in the presence of large glycans, which is caused by the glycan at position N231. Error bars represent SEM derived from three independent measurements. (e) Effect of CD22 glycoforms on epratuzumab binding thermodynamics. Similar to what was observed in biolayer interferometry, epratuzumab Fab binds with lower affinity to CD22 with large glycans. (f) Calculated K_D, ΔH and –TΔS values of epratuzumab Fab binding to $CD22_{20\text{-}687}$F, $CD22_{20\text{-}687}$S and $dCD22_{20\text{-}687}$. Error bars represent SEM derived from three independent measurements. (g) Epratuzumab IgG (teal) binds at an angle that facilitates extensive crosslinking of CD22 nanoclusters. Crystal structures of $CD22_{20\text{-}330,5A}$ (grey surface) and $CD22_{20\text{-}330,4Q}$+epratuzumab Fab (grey and teal surface, respectively) are fit into 3D reconstructions calculated from negative stain EM of $CD22_{20\text{-}687}$ and $CD22_{20\text{-}687}$+epratuzumab, respectively. Epratuzumab IgG (teal) was modelled based on the crystal structure of human anti-HIV-1 IgG (1HZH) [11]. Modelled N-linked glycans are shown as a blue surface.

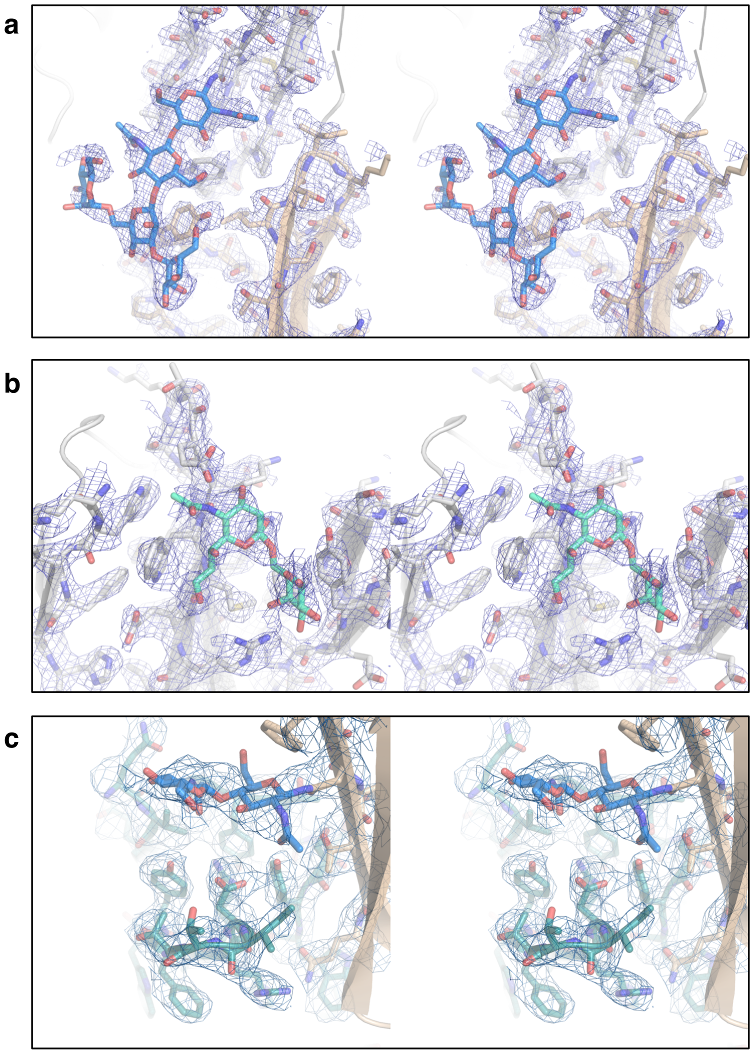

Supplementary Figure 8. Representative electron density for reported crystal structures. Composite omit map contoured at 1σ is shown in stereo rendering for **a)** the CD22 N101 glycan and surrounding residues at the CD22 d1-d2 interface; **b)** the α2-6 sialyllactose ligand and CD22 binding site residues; and **c)** epratuzumab-CD22 residues involved at the antibody-antigen interface.

Supplementary Table 1. Interactions between α2-6 sialyllactose and CD22.

	**CD22 Residue**	**BSA (Å^2)**
	Lys 23	8
	Trp 24	3
	Tyr 64	60
	Lys 66	10
	Ser 69	1
	Phe 71	22
HS	Arg 120	13
H	Glu 126	39
H	Lys 127	20
H	Trp 128	47
H	Met 129	23
H	Arg 131	29
	**Total**	**275**

Left column: H (hydrogen-bond), S (salt bridge). Buried surface area (BSA) in Å^2.

Supplementary Table 2. SAXS data-collection and scattering-derived parameters.

	$CD22_{20\text{-}687}$	$CD22_{20\text{-}687}$ + α2-6 sialyllactose
**Data-Collection parameters**		
Concentration (mg ml^{-1})	5	5
**Structural parameters**		
I_0 (cm^{-1}) [from Guinier]	0.4	0.3
R_g (Å) [from Guinier]	80.1	81.0
I_0 (cm^{-1}) [from P(r)]	0.4	0.3
R_g (Å) [from P(r)]	84.3	84.8
D_{max} (Å)	305.7	298.0
Porod volume (nm^3)	296.2	306.4
MW (kDa)	90	90
MW calculated with Scatter (kDa)	80.8	80.0
Ab initio analysis	DAMMIF	DAMMIF
χ^2 of *ab initio* models	0.2-0.3	0.1-0.2

Supplementary Table 3. Recognition determinants of epratuzumab Fab binding to CD22 ECD.

	CD22 residue	BSA (Å^2)
	Pro 142	6
	His 144	11
H	Ile 145	7
	Leu 147	14
	Pro 148	1
	Pro 149	17
	Glu 150	1
	Leu 178	22
	Glu 179	96
	Gly 180	16
	Val 181	18
	Arg 184	6
	Trp 210	7
H	Ser 211	28
	His 212	3
S	His 213	65
H	Gly 214	29
	Lys 215	75
	Ile 216	65
	Thr 218	5
HS	Asp 232	25
H	Thr 233	51
	Val 234	34
H	Gln 235	122
	Leu 236	1
	Asn 237	82
	Lys 239	15
	Glu 245	37
	Lys 247	24
	Cys 265	1
	Glu 266	41
H	Val 267	44
	Ser 268	32
	Ser 269	17
	Ser 270	5
H	Asn 271	21
	Pro 272	13
	Glu 273	87
H	Tyr 274	70
	Thr 275	4
	Phe 292	14
	NAG1231	27
	NAG1232	49
	**Total**	**1,308**
	**Epratuzumab HC residue**	**BSA (Å^2)**
	Gln^{H1}	1
	Val^{H2}	45
	Gly^{H26}	9
	Tyr^{H27}	3
	Thr^{H28}	13
	Thr^{H30}	2
H	Ser^{H31}	55
	Tyr^{H32}	48
H	Trp^{H33}	99
	His^{H35}	2
	Trp^{H47}	5

	Asn^{H52}	12
HS	Arg^{H53}	96
	Asn^{H54}	31
H	Tyr^{H56}	75
S	Glu^{H58}	15
	Tyr^{H59}	15
H	Gln^{H61}	47
	Lys^{H64}	33
HS	Arg^{H95}	66
	Asp^{H96}	7
	Ile^{H97}	111
	Tyr^{H105}	8
	**Total**	**798**
	**Epratuzumab LC residue**	**BSA (Å^2)**
H	Asp^{L1}	103
	Ile^{L2}	22
	Ser^{L26}	40
H	Gln^{L27}	76
	Ser^{L27A}	9
H	Tyr^{L27D}	81
	Ser^{L27E}	9
	Ala^{L27F}	18
	Asn^{L28}	35
	His^{L29}	1
	Tyr^{L32}	22
	Trp^{L50}	28
	Tyr^{L91}	2
H	Leu^{L92}	44
	Ser^{L93}	75
	Ser^{L94}	12
	Trp^{L96}	5
	**Total**	**582**

Left column: H (hydrogen-bond), S (salt bridge). Buried surface area (BSA) in Å^2.

Supplementary Table 4. Codon optimized DNA sequences.

$CD22_{20\text{-}687}$	GACTCATCTAAATGGGTCTTCGAGCATCCTGAAACACTGTATGCCTGGGA<br>GGGCGCTTGCGTCTGGATTCCCTGTACTTATCGGGCCCTGGACGGGGATC<br>TGGAGTCTTTCATCCTGTTTCACAACCCAGAATACAACAAGAATACTAGC<br>AAATTCGACGGGACCAGACTGTATGAGAGTACAAAGGATGGAAAAGTCC<br>CCTCAGAACAGAAGAGGGTGCAGTTTCTGGGCGACAAGAACAAAAATTG<br>CACACTGAGTATCCACCCTGTGCATCTGAACGATTCAGGCCAGCTGGGGC<br>TGAGGATGGAAAGCAAGACTGAGAAATGGATGGAAAGAATCCACCTGAA<br>TGTGAGCGAGCGGCCTTTCCCCCCTCATATCCAGCTGCCACCCGAGATTC<br>AGGAAAGCCAGGAGGTGACCCTGACATGCCTGCTGAATTTTTCCTGTTAC<br>GGCTATCCAATCCAGCTGCAGTGGCTGCTGGAGGGGGTCCCCATGCGACA<br>GGCAGCTGTGACTAGTACCTCACTGACTATCAAAAGCGTGTTCACCCGAT<br>CCGAACTGAAGTTTTCCCCCCAGTGGTCTCACCACGGCAAGATTGTGACC<br>TGCCAGCTGCAGGACGCCGATGGAAAGTTCCTGTCCAACGACACCGTGCA<br>GCTGAATGTCAAGCACACACCCAAACTGGAGATCAAGGTCACTCCTTCCG<br>ACGCTATTGTGAGAGAAGGCGATTCTGTCACAATGACTTGTGAGGTGAGC<br>AGCAGCAACCCTGAATACACCACAGTGAGTTGGCTGAAAGATGGCACTT<br>CACTGAAGAAACAGAACACCTTTACACTGAATCTGAGGGAGGTGACCAA<br>AGACCAGAGCGGGAAGTACTGCTGTCAGGTGTCCAATGATGTCGGCCCA<br>GGGCGCTCTGAGGAAGTGTTCCTGCAGGTCCAGTATGCCCCAGAACCCAG<br>CACCGTGCAGATCCTGCACTCACCCGCTGTGGAGGGCAGCCAGGTCGAAT<br>TTCTGTGCATGTCCCTGGCCAACCCTCTGCCAACCAATTACACATGGTATC<br>ATAACGGGAAGGAGATGCAGGGACGAACAGAGGAAAAAGTCCACATCC<br>CAAAGATTCTGCCCTGGCATGCAGGAACCTACTCTTGCGTGGCCGAGAAT<br>ATTCTGGGAACAGGCCAGCGAGGACCTGGAGCAGAACTGGACGTGCAGT<br>ATCCTCCAAAGAAAGTGACTACCGTCATCCAGAACCCCATGCCTATTCGG<br>GAGGGCGATACTGTCACCCTGAGTTGCAACTACAATAGTTCAAATCCATC<br>CGTGACCAGATATGAATGGAAACCTCACGGGGCTTGGGAGGAACCAAGC<br>CTGGGAGTCCTGAAGATCCAGAACGTGGGCTGGGACAATACAACTATTG<br>CATGCGCAGCCTGTAACAGTTGGTGCTCATGGGCCAGCCCAGTGGCTCTG<br>AATGTCCAGTACGCACCCCGGGACGTGAGGGTCCGCAAGATCAAACCTCT<br>GAGTGAGATTCACTCAGGCAACAGCGTCTCCCTGCAGTGTGATTTCAGCT<br>CCTCTCATCCAAAGGAGGTGCAGTTCTTTTGGGAGAAGAACGGAAGACTG<br>CTGGGCAAGGAGAGCCAGCTGAATTTTGACTCTATCAGTCCCGAAGATGC<br>TGGATCATACAGCTGTTGGGTGAACAATTCCATTGGCCAGACAGCTTCTA<br>AAGCATGGACTCTGGAGGTGCTGTATGCACCTCGGAGACTGAGGGTCTCC<br>ATGTCTCCAGGAGATCAGGTCATGGAGGGCAAGAGCGCCACCCTGACTT<br>GCGAATCCGACGCAAACCCCCCCGTGAGCCATTACACCTGGTTCGATTGG<br>AACAATCAGTCTCTGCCCTATCACAGTCAGAAACTGCGCCTGGAGCCTGT<br>GAAGGTCCAGCATTCTGGCGCCTATTGGTGTCAGGGCACAAATAGCGTGG<br>GGAAGGGCAGAAGCCCCCTGAGCACCCTGACAGTCTACTACAGCCCTGA<br>AACTATCGGAAGGCGG
$CD22_{20\text{-}330,5A}$	GACAGCAGCAAATGGGTGTTCGAGCACCCCGAGACACTGTACGCCTGGG<br>AAGGCGCCTGCGTGTGGATTCCCTGTACCTACAGAGCCCTGGACGGCGAC<br>CTGGAATCCTTCATCCTGTTCCACAACCCCGAGTACAACAAGGCCACCAG<br>CAAGTTCGACGGCACCCGGCTGTACGAGAGCACCAAGGATGGCAAGGTG<br>CCCAGCGAGCAGAAACGGGTGCAGTTCCTGGGCGACAAGAACAAGAATT<br>GCACCCTGAGCATCCACCCCGTGCACCTGGCCGATTCTGGACAGCTGGGC<br>CTGCGGATGGAAAGCAAGACCGAGAAGTGGATGGAACGCATCCATCTGG<br>CCGTGTCCGAGAGGCCCTTCCCACCTCACATTCAGCTGCCCCCCGAGATC<br>CAGGAATCCCAGGAAGTGACCCTGACCTGCCTGCTGGCCTTCAGCTGCTA<br>CGGCTACCCCATCCAGCTGCAGTGGCTGCTGGAAGGCGTGCCCATGAGAC

	AGGCCGCCGTGACAAGCACCAGCCTGACCATCAAGAGCGTGTTCACCAG AAGCGAGCTGAAGTTCAGCCCCCAGTGGTCCCACCACGGCAAGATCGTG ACATGCCAGCTGCAGGACGCCGACGGCAAGTTCCTGAGCGCCGATACAG TGCAGCTGAACGTGAAGCACACCCCCAAGCTGGAAATCAAAGTGACCCC CAGCGACGCCATCGTGCGCGAGGGCGATAGCGTGACCATGACATGCGAG GTGTCCAGCAGCAATCCTGAGTACACCACCGTGTCCTGGCTGAAGGACGG CACCTCCCTGAAGAAGCAGAACACCTTCACCCTGAACCTGCGCGAAGTGA CCAAGGACCAGAGCGGCAAGTACTGCTGCCAGGTGTCCAACGATGTGGG CCCTGGCAGATCCGAAGAGGTGTTCCTGCAGGTGCAGTATGCCGGC
$CD22_{20\text{-}330,4Q}$	GACAGCAGCAAATGGGTGTTCGAGCACCCCGAGACACTGTACGCCTGGG AAGGCGCCTGCGTGTGGATTCCCTGTACCTACAGAGCCCTGGACGGCGAC CTGGAATCCTTCATCCTGTTCCACAACCCCGAGTACAACAAGCAGACCAG CAAGTTCGACGGCACCCGGCTGTACGAGAGCACCAAGGATGGCAAGGTG CCCAGCGAGCAGAAACGGGTGCAGTTCCTGGGCGACAAGAACAAGAATT GCACCCTGAGCATCCACCCGGTGCATCTGCAGGATTCTGGCCAGCTGGGC CTGCGGATGGAAAGCAAGACCGAGAAGTGGATGGAACGCATCCATCTGC AGGTGTCCGAGCGGCCCTTCCCACCTCACATTCAGCTGCCCCCCGAGATC CAGGAATCCCAGGAAGTGACCCTGACCTGCCTGCTGCAGTTCAGCTGCTA CGGCTACCCCATCCAGCTGCAGTGGCTGCTGGAAGGCGTGCCCATGAGAC AGGCCGCCGTGACAAGCACCAGCCTGACCATCAAGAGCGTGTTCACCAG AAGCGAGCTGAAGTTCAGCCCCCAGTGGTCCCACCACGGCAAGATCGTG ACATGCCAGCTGCAGGACGCCGACGGCAAGTTCCTGAGCAATGATACCG TGCAGCTGAACGTGAAGCACACCCCCAAGCTGGAAATCAAAGTGACCCC CAGCGACGCCATCGTGCGCGAGGGCGATAGCGTGACCATGACATGCGAG GTGTCCAGCAGCAATCCTGAGTACACCACCGTGTCCTGGCTGAAGGACGG CACCTCCCTGAAGAAGCAGAACACCTTCACCCTGAACCTGCGCGAAGTGA CCAAGGACCAGAGCGGCAAGTACTGCTGCCAGGTGTCCAACGATGTGGG CCCTGGCAGATCCGAAGAGGTGTTCCTGCAGGTGCAGTATGCCGGC

Supplementary Table 5. Primer sequences.

$CD22_{20\text{-}687}$ N67Q Fw	5'-ACAACCCAGAATACAACAAGCAGACTAGCAAATTCGACGGGAC-3'
$CD22_{20\text{-}687}$ N67Q Rv	5'-GTCCCGTCGAATTTGCTAGTCTGCTTGTTGTATTCTGGGTTGT-3'
$CD22_{20\text{-}687}$ N101Q Fw	5'-GCAGTTTCTGGGCGACAAGAACAAACAGTGCACACTGAGTA-3'
$CD22_{20\text{-}687}$ N101Q Rv	5'-TACTCAGTGTGCACTGTTTGTTCTTGTCGCCCAGAAACTGC-3'
$CD22_{20\text{-}687}$ N112Q Fw	5'-CACCCTGTGCATCTGCAGGATTCAGGCCAGCTG-3'
$CD22_{20\text{-}687}$ N112Q Rv	5'-CAGCTGGCCTGAATCCTGCAGATGCACAGGGTG-3'
$CD22_{20\text{-}687}$ N135Q Fw	5'-GGAAAGAATCCACCTGCAGGTGAGCGAGCGGCCTT-3'
$CD22_{20\text{-}687}$ N135Q Rv	5'-AAGGCCGCTCGCTCACCTGCAGGTGGATTCTTTCC-3'
$CD22_{20\text{-}687}$ N164Q Fw	5'-GACCCTGACATGCCTGCTGCAGTTTTCCTGTTACGGCTATC-3'
$CD22_{20\text{-}687}$ N164Q Rv	5'-GATAGCCGTAACAGGAAAACTGCAGCAGGCATGTCAGGGTC-3'
$CD22_{20\text{-}687}$ N231Q Fw	5'-GGAAAGTTCCTGTCCCAGGACACCGTGCAGCTG-3'
$CD22_{20\text{-}687}$ N231Q Rv	5'-CAGCTGCACGGTGTCCTGGGACAGGAACTTTCC-3'
CD22 Fw	5’-TTTTACCGGTGACTCATCTAAATGGGTCTTC-3’
$CD22_{20\text{-}330}$ Rv	5’-TTTTGGTACCGGCATACTGGACCTGCAGGAAC-3’
$CD22_{20\text{-}504}$ Rv	5’-TTTTGGTACCTGCGTACTGGACATTCAGAGCCAC-3’
$CD22_{20\text{-}592}$ Rv	5’-TTTTGGTACCTGCATACAGCACCTCCAGAGTCCA-3’

References

1. Gupta, R., Jung, E. & Brunak, S. NetNGlyc: Prediction of N-glycosylation sites in human proteins. (2004).
2. Pronker, M. F. *et al.* Structural basis of myelin-associated glycoprotein adhesion and signalling. *Nat. Commun.* **7,** 1–13 (2016).
3. Zhuravleva, M. A., Trandem, K. & Sun, P. D. Structural implications of Siglec-5-mediated sialoglycan recognition. *J. Mol. Biol.* **375,** 437–447 (2008).
4. May, A. P., Robinson, R. C., Vinson, M., Crocker, P. R. & Jones, E. Y. Crystal structure of the N-terminal domain of sialoadhesin in complex with 3' sialyllactose at 1.85 Å resolution. *Mol. Cell* **1,** 719–728 (1998).
5. Alphey, M. S., Attrill, H., Crocker, P. R. & van Aalten, D. M. F. High resolution crystal structures of Siglec-7. Insights into ligand specificity in the Siglec family. *J. Biol. Chem.* **278,** 3372–3377 (2003).
6. Attrill, H. *et al.* Siglec-7 undergoes a major conformational change when complexed with the (2,8)-disialylganglioside GT1b. *J. Biol. Chem.* **281,** 32774–32783 (2006).
7. Pröpster, J. M. *et al.* Structural basis for sulfation-dependent self-glycan recognition by the human immune-inhibitory receptor Siglec-8. *Proc. Natl. Acad. Sci.* **113,** E4170–E4179 (2016).
8. Chemical Computing Group Inc. Molecular Operating Environment (MOE). *Sci. Comput. Instrum.* **22,** 32 (2004).
9. Svergun, D. I. Restoring low resolution structure of biological macromolecules from solution scattering using simulated annealing. *Biophys. J.* **76,** 2879–2886 (1999).
10. Punjani, A., Rubinstein, J. L., Fleet, D. J. & Brubaker, M. A. cryoSPARC: algorithms for rapid unsupervised cryo-EM structure determination. *Nat. Methods* **14,** 290–296 (2017).
11. Saphire, E. O. *et al.* Crystal structure of a neutralizing human IgG against HIV-1: a template for vaccine design. *Science* **293,** 1155–1159 (2001).
